# Supplementary figures and images for: Clearance of protein aggregates during cell division
Source: eLife. 2025 Jun 6;13:RP96675. doi: 10.7554/eLife.96675 (PMC12143881; doi:10.7554/eLife.96675)

**Figure 1- source data 1**

**Original membranes corresponding to Figure 1C**

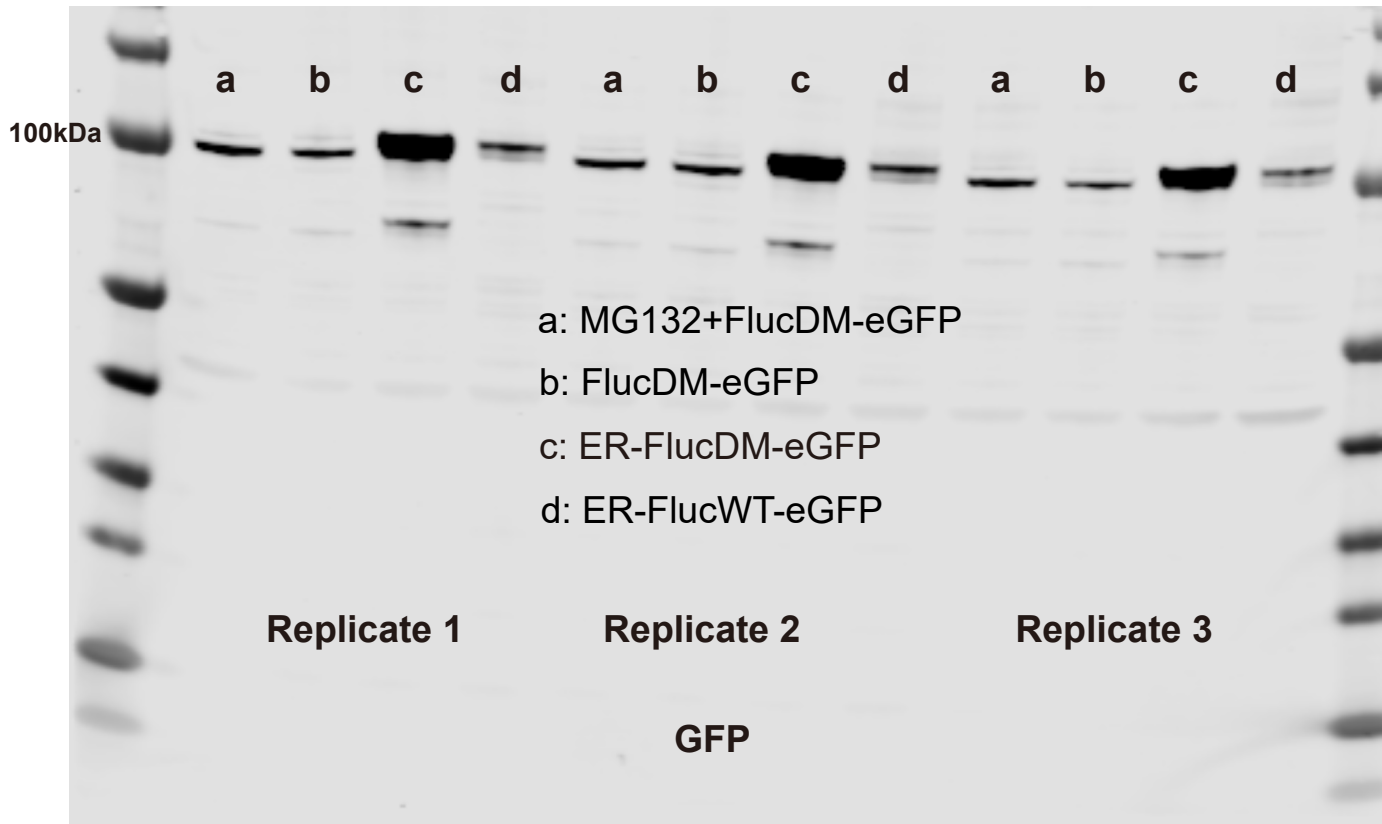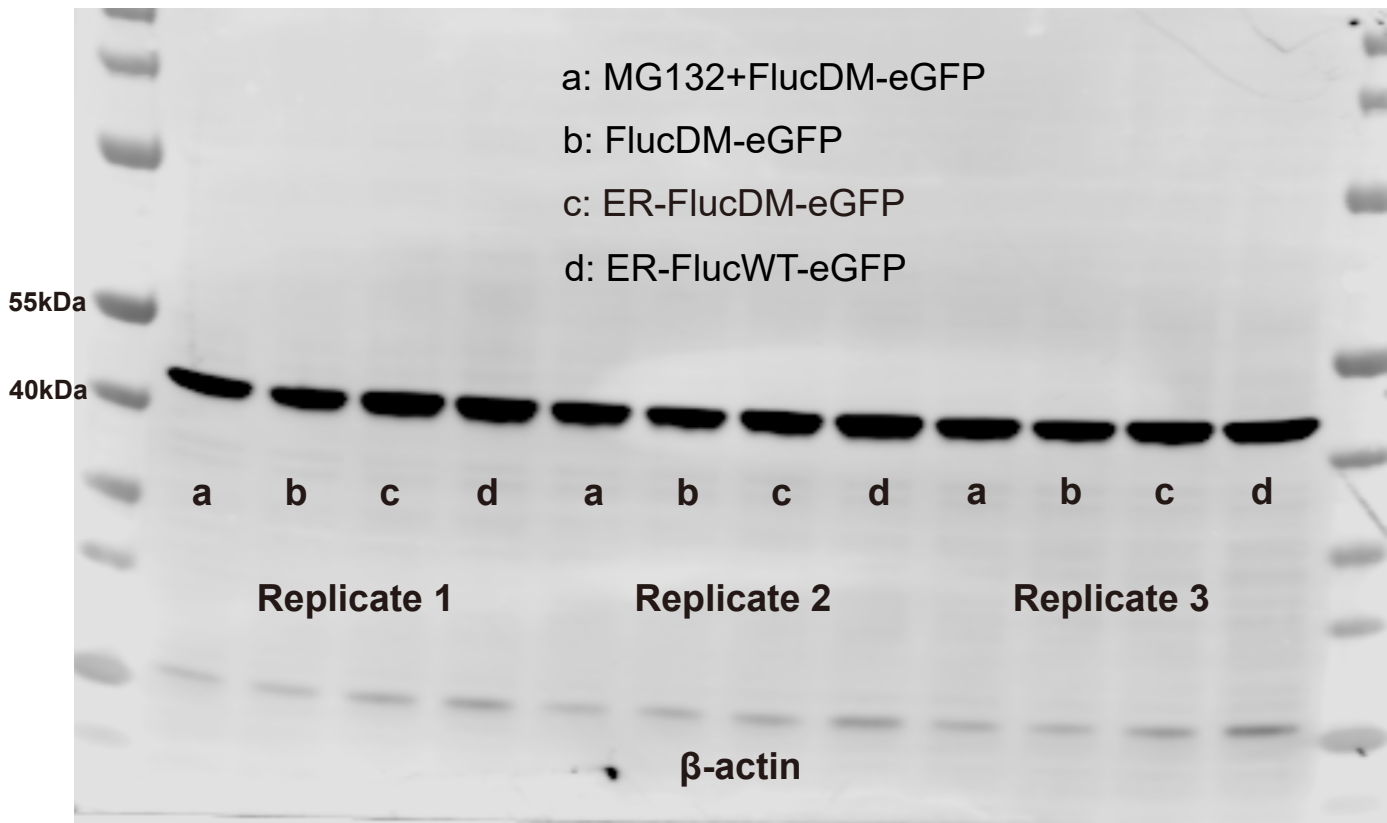

Supplement: Figure 1—source data 1. [file elife-96675-fig1-data1.zip › Figure 1- source data 1/Figure 1- source data 1.pdf]

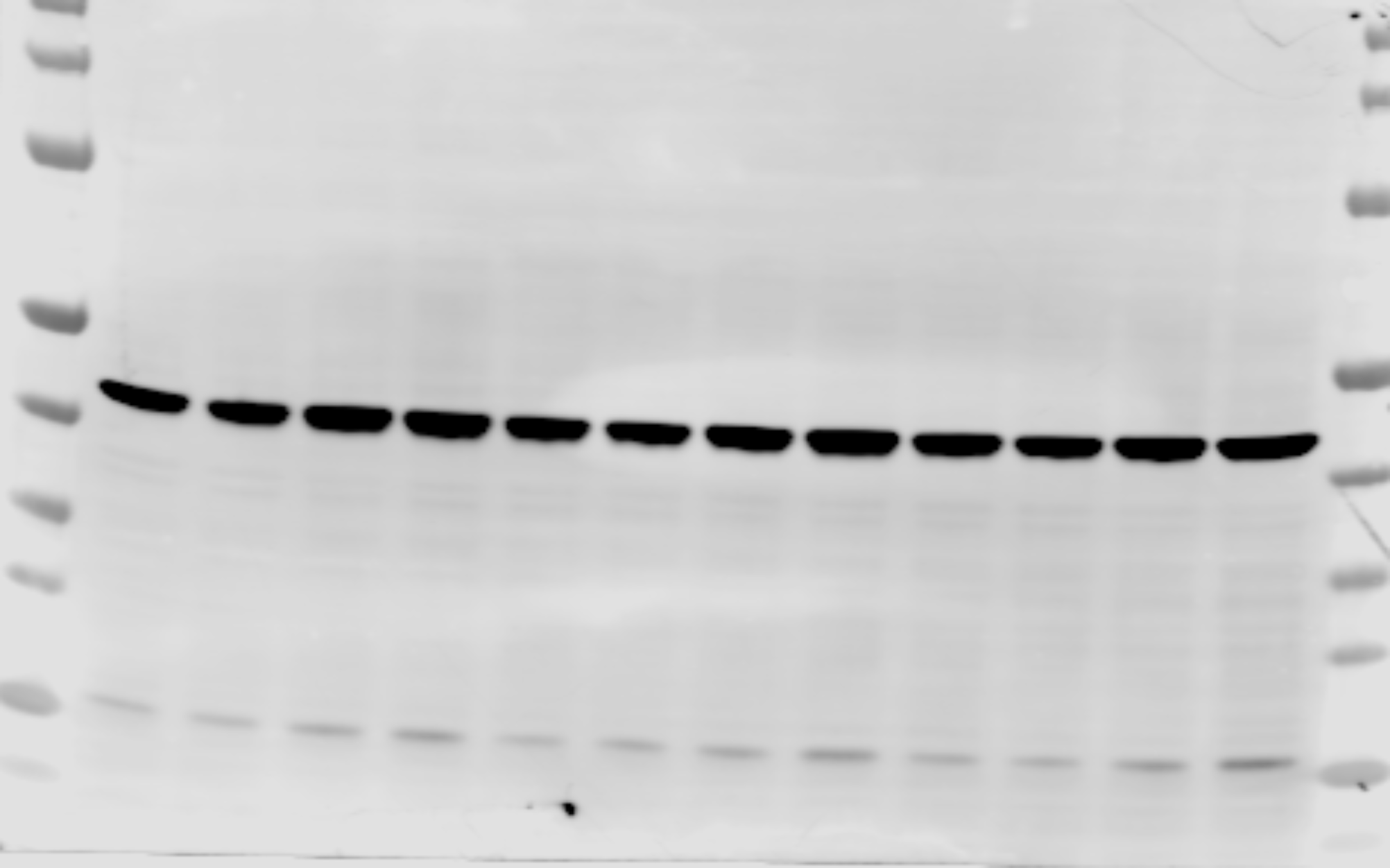

Supplement: Figure 1—source data 2. [file elife-96675-fig1-data2.zip › Figure 1- source data 2/Luci_b-actin.tif]

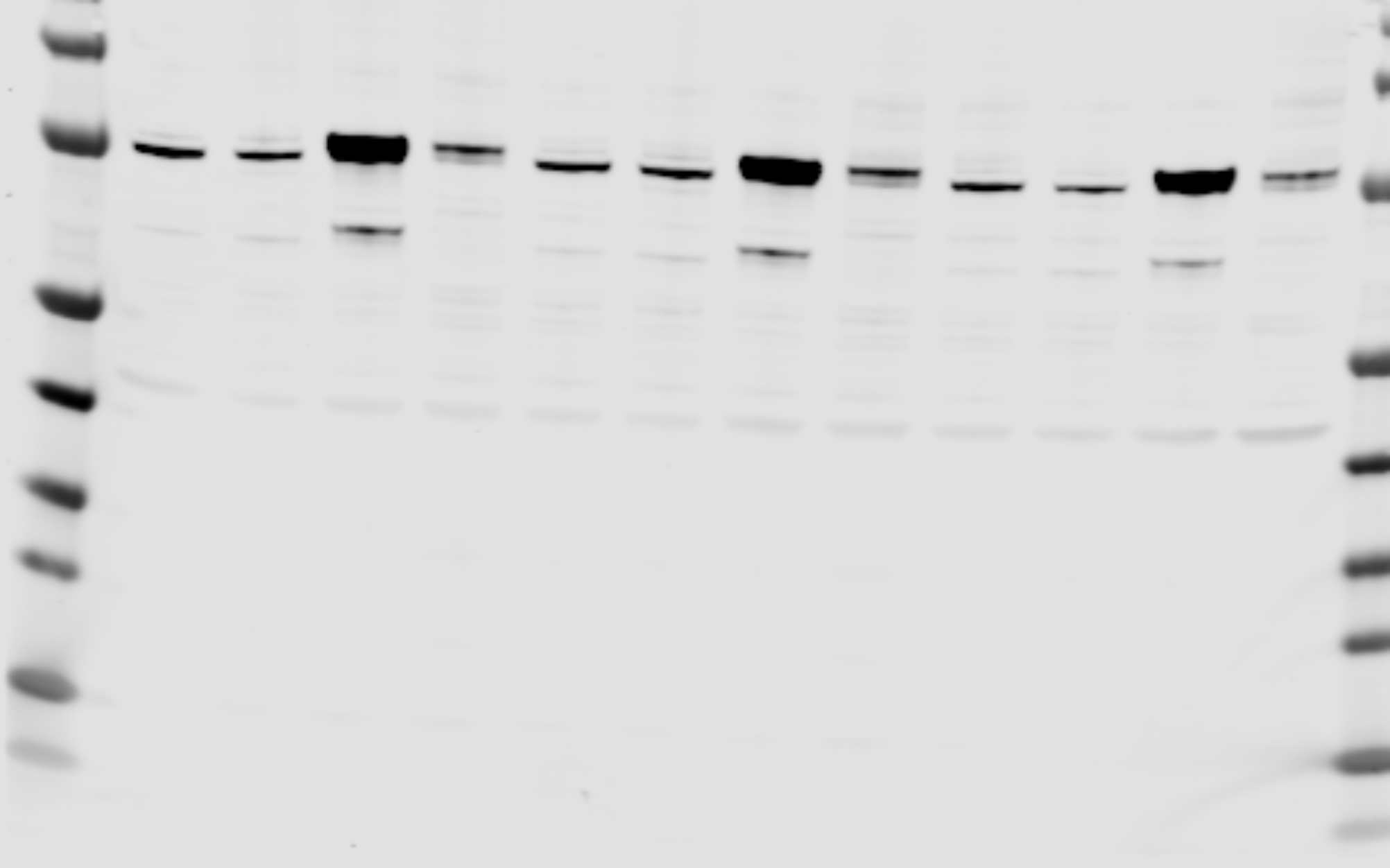

Supplement: Figure 1—source data 2. [file elife-96675-fig1-data2.zip › Figure 1- source data 2/Luci_GFP.tif]

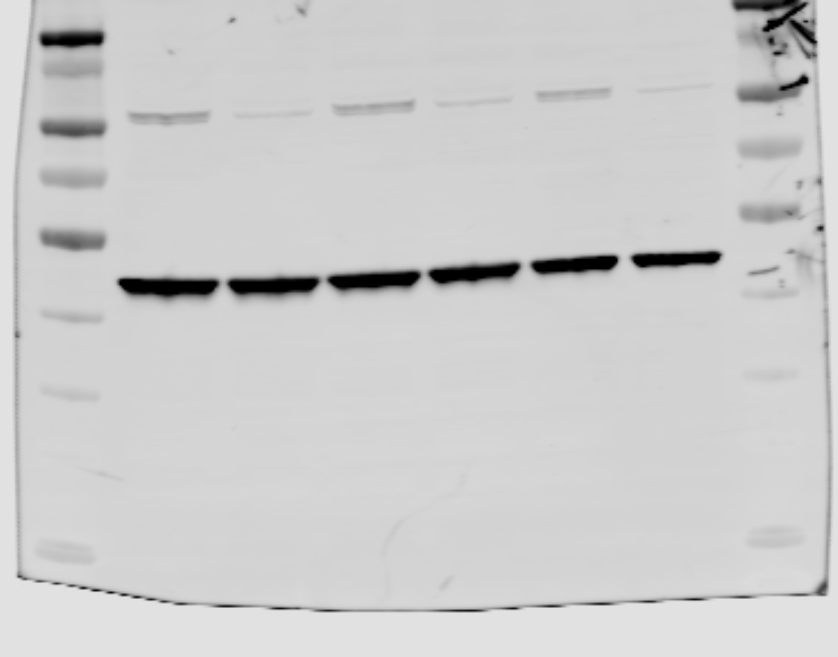

Supplement: Figure 1—figure supplement 1—source data 2. [file elife-96675-fig1-figsupp1-data2.zip › Figure 1-figure supplement 1-source data 2/NLS_ER_b-actin.tif]

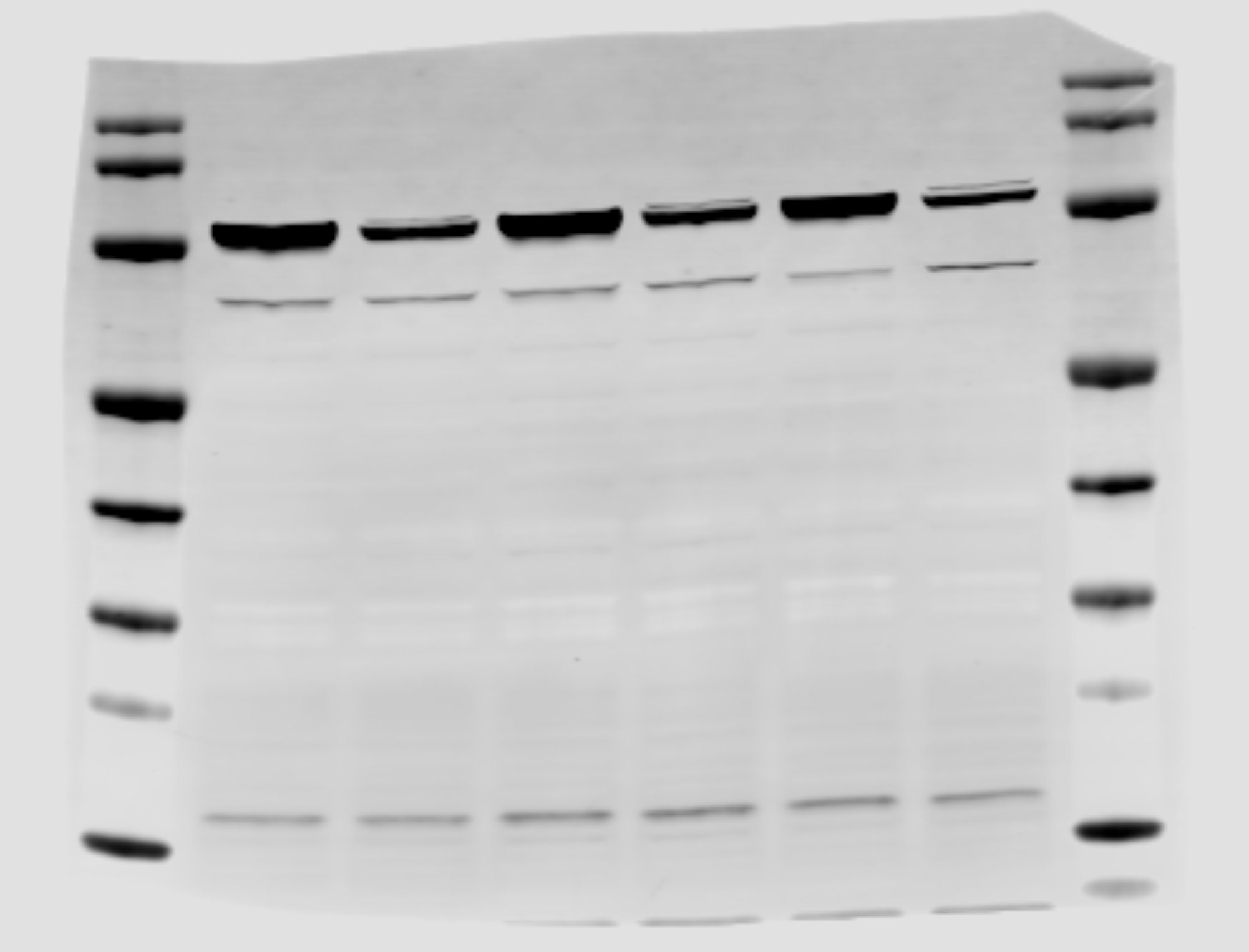

Supplement: Figure 1—figure supplement 1—source data 2. [file elife-96675-fig1-figsupp1-data2.zip › Figure 1-figure supplement 1-source data 2/NLS_ER_GFP.tif]

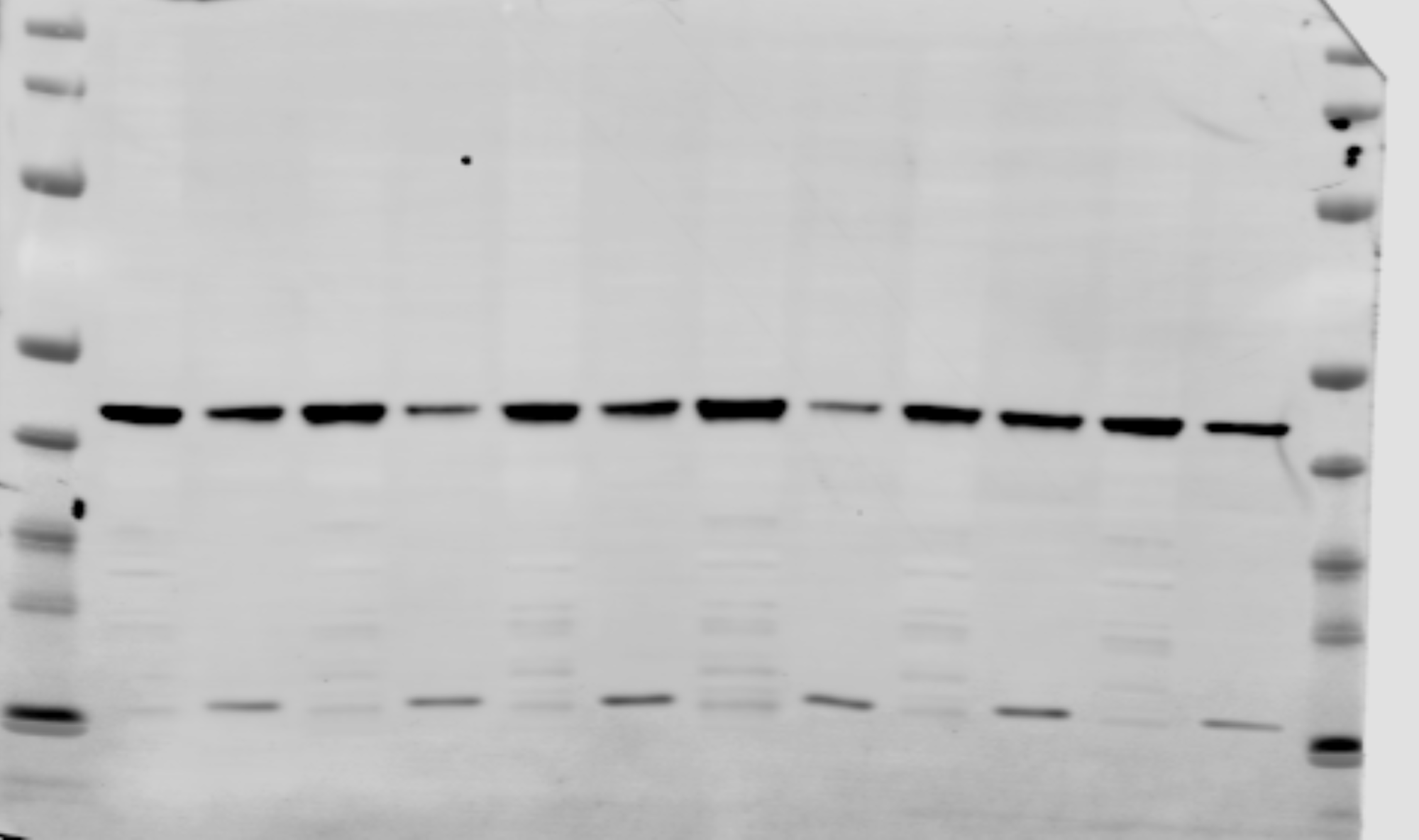

Supplement: Figure 1—figure supplement 1—source data 2. [file elife-96675-fig1-figsupp1-data2.zip › Figure 1-figure supplement 1-source data 2/solubility_b-actin.tif]

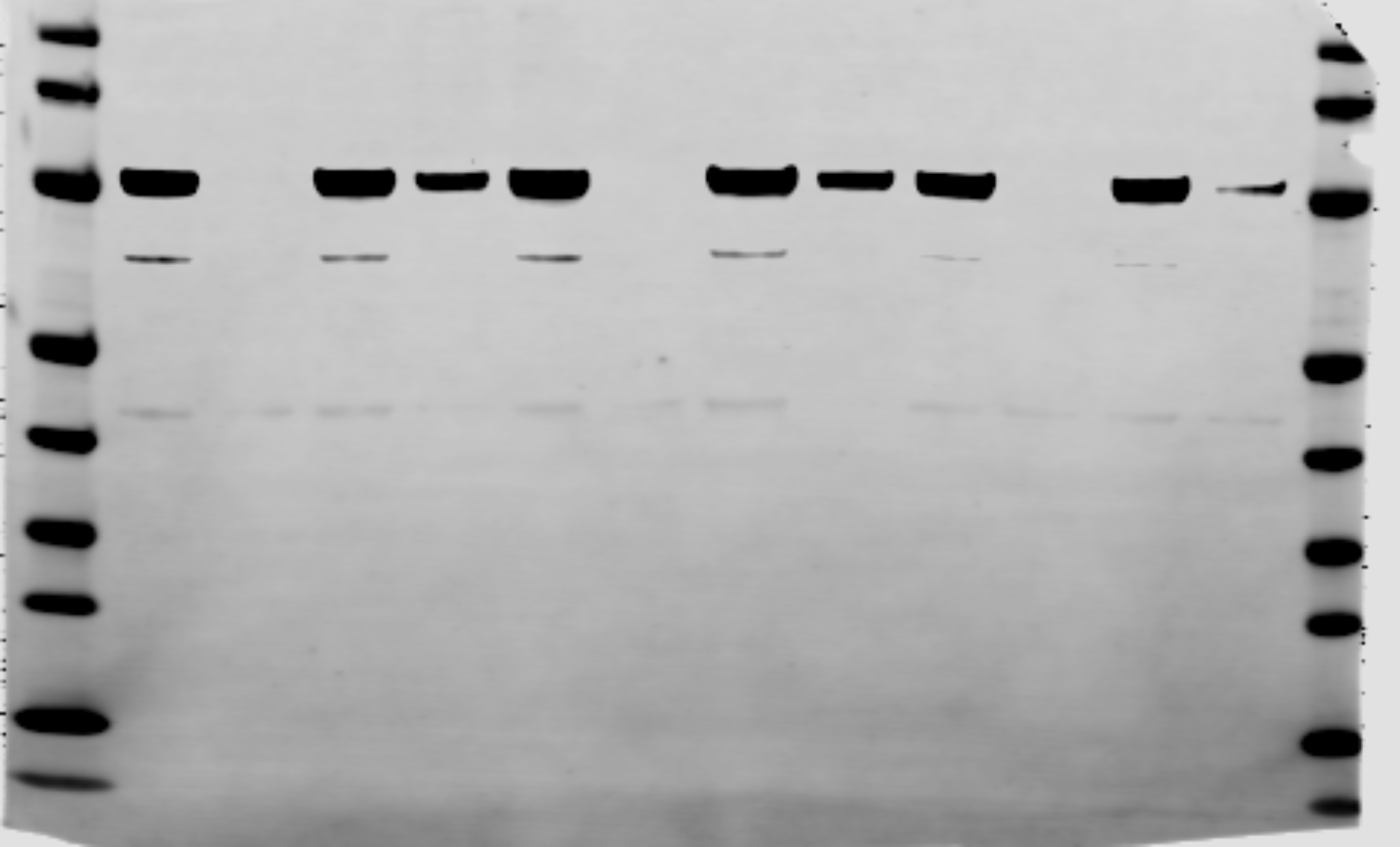

Supplement: Figure 1—figure supplement 1—source data 2. [file elife-96675-fig1-figsupp1-data2.zip › Figure 1-figure supplement 1-source data 2/solubility_GFP.tif]

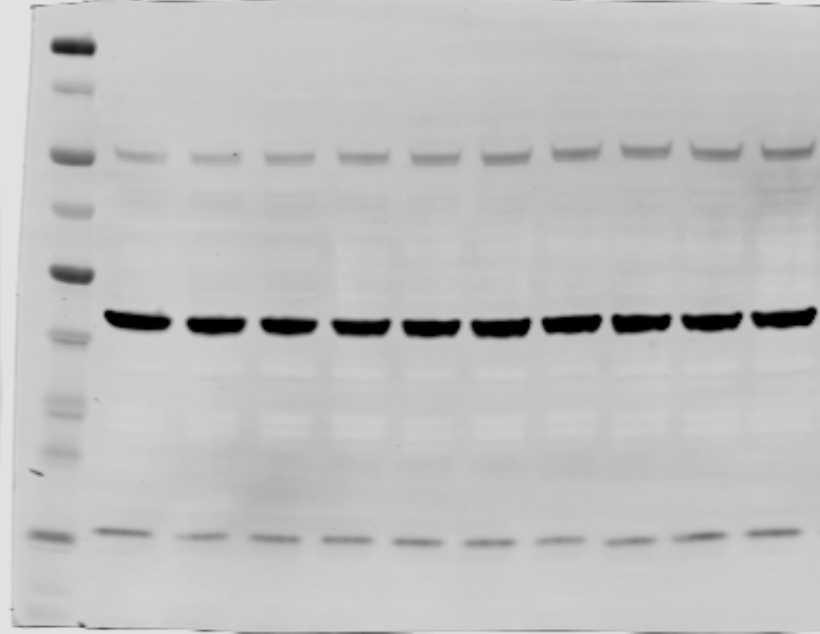

Supplement: Figure 6—figure supplement 2—source data 1. [file elife-96675-fig6-figsupp2-data1.zip › Figure 7-figure supplement 1-source data 1/CHX_chase_b-actin.tif]

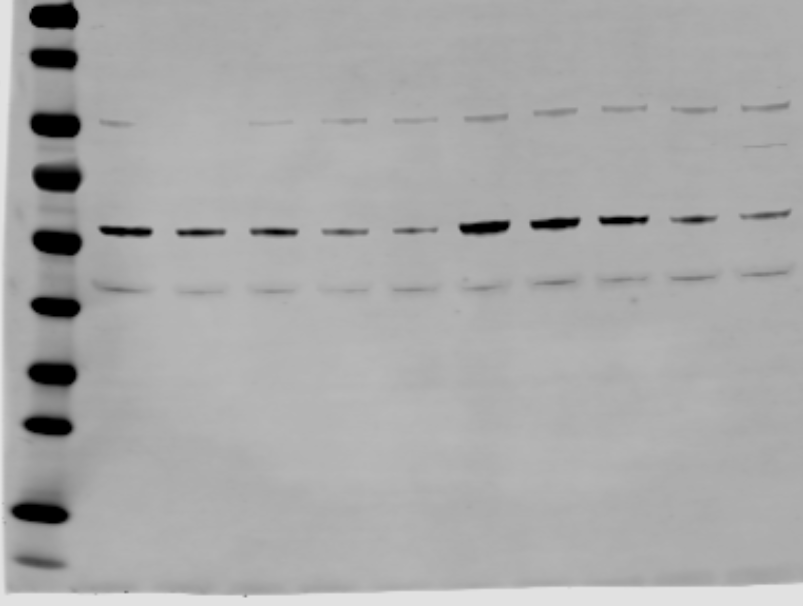

Supplement: Figure 6—figure supplement 2—source data 1. [file elife-96675-fig6-figsupp2-data1.zip › Figure 7-figure supplement 1-source data 1/CHX_chase_cyclinB.tif]

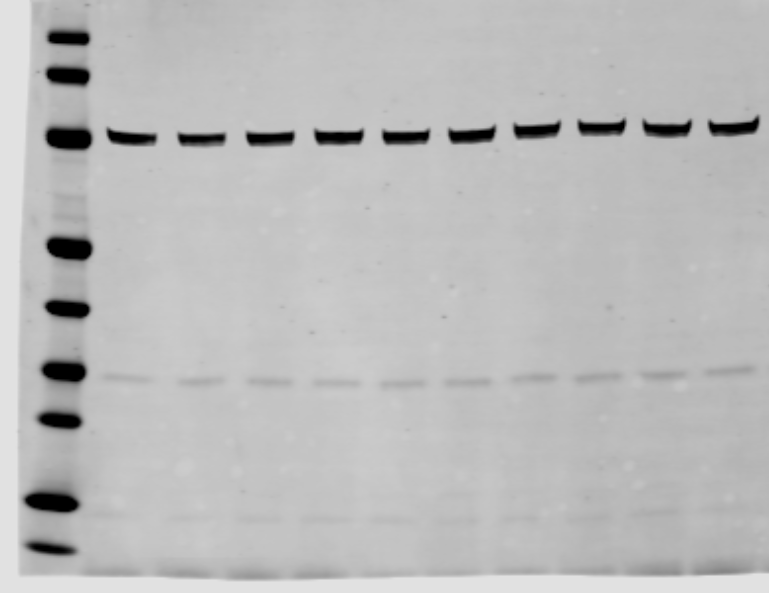

Supplement: Figure 6—figure supplement 2—source data 1. [file elife-96675-fig6-figsupp2-data1.zip › Figure 7-figure supplement 1-source data 1/CHX_chase_GFP.tif]

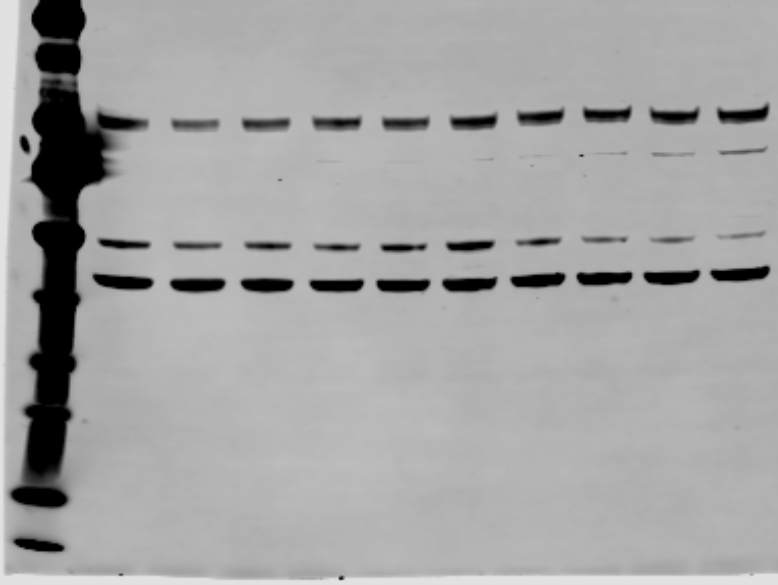

Supplement: Figure 6—figure supplement 2—source data 1. [file elife-96675-fig6-figsupp2-data1.zip › Figure 7-figure supplement 1-source data 1/CHX_chase_p53.tif]

## Figure 7-figure supplement 1-source data 1

### Original membranes corresponding to Figure S7

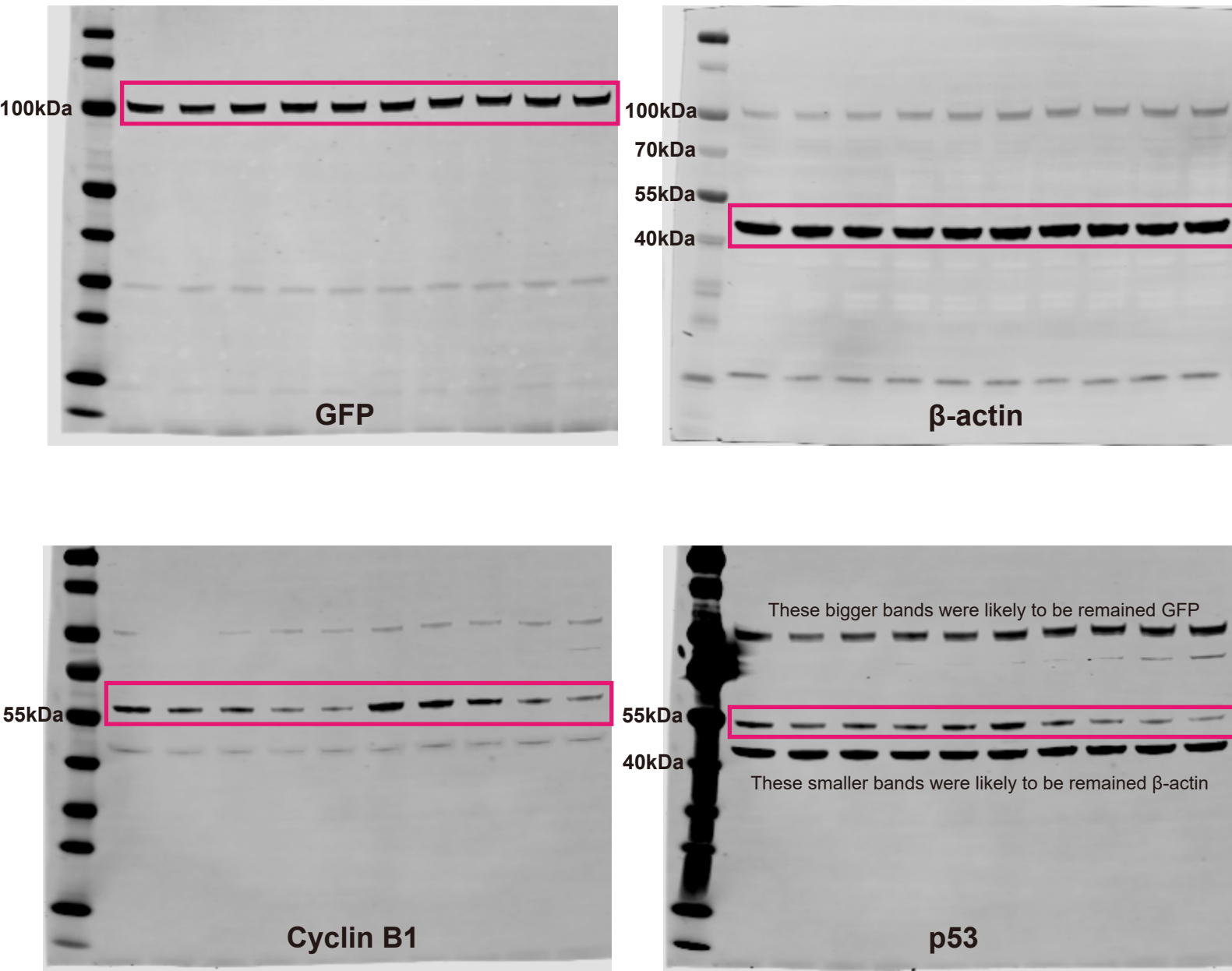

Supplement: Figure 6—figure supplement 2—source data 1. [file elife-96675-fig6-figsupp2-data1.zip › Figure 7-figure supplement 1-source data 1/Figure 7-figure supplement 1-source data 1.pdf]
