# Supplementary material for: Clearance of protein aggregates during cell division
Source: eLife. 2025 Jun 6;13:RP96675. doi: 10.7554/eLife.96675 (PMC12143881; doi:10.7554/eLife.96675)

Figure 1-figure supplement 1-source data 1

Original membranes corresponding to Figure S1C and S1D

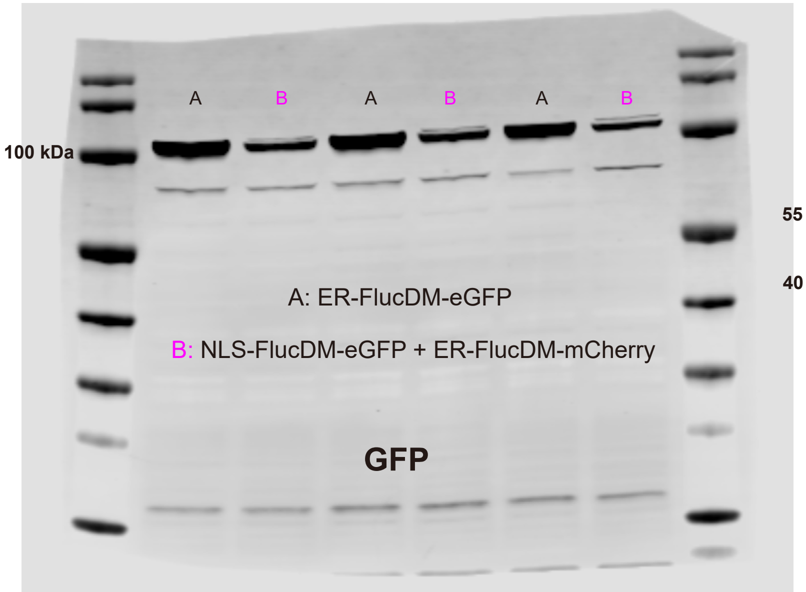

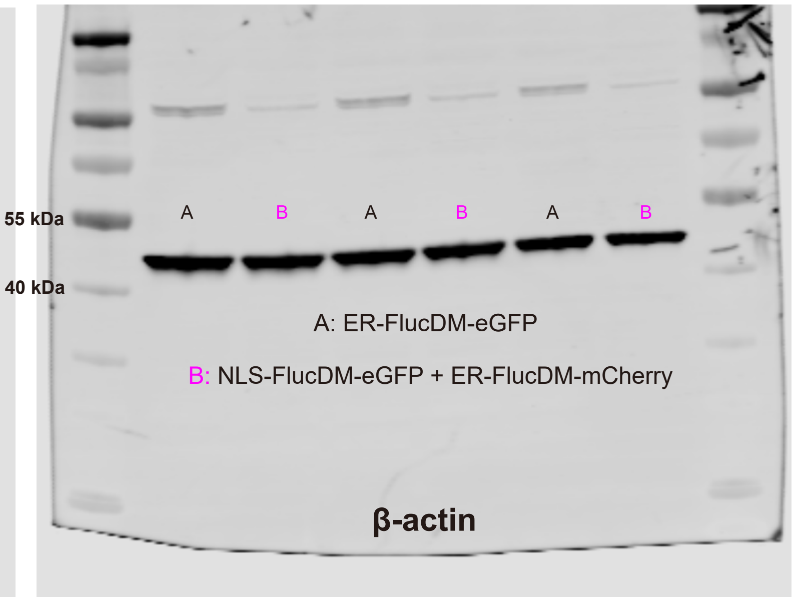

Original membranes corresponding to Figure S1G

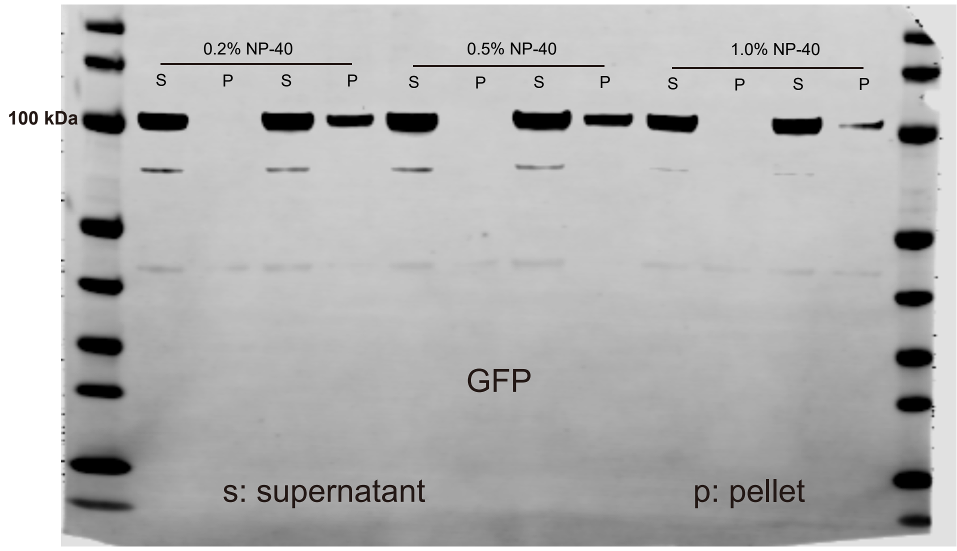

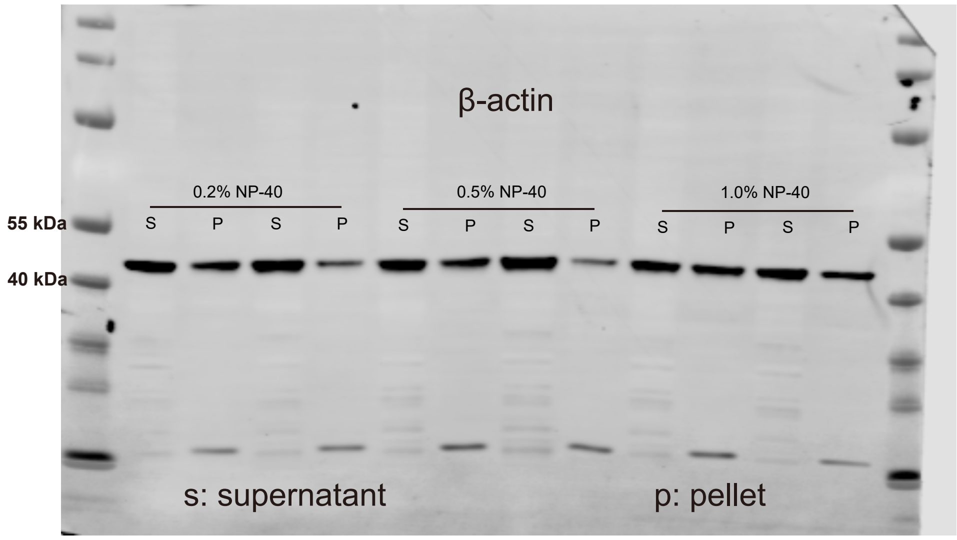

Supplement: Figure 1—figure supplement 1—source data 1. [file elife-96675-fig1-figsupp1-data1.zip › Figure 1-figure supplement 1-source data 1/Figure 1-figure supplement 1-source data 1.pdf]
